# Determinants of Serum Iron Profile in Non-Anemic Pregnant Women at Delivery: A Cross-Sectional Study at Baharloo Hospital (2022-2023): Iron Deficiency Among Non-anemic Pregnant Women

**DOI:** 10.31661/gmj.v14i.3895

**Published:** 2025-09-12

**Authors:** Seyedeh Noushin Ghalandarpoor-Atta, Seyedeh Mojgan Ghalandarpoor-Attar, Seyyed Mohammad Mahdi Tayebi Tafreshi, Asghar Ghorbani

**Affiliations:** ^1^ Obstetrics and Gynecology Department, Baqiyatallah Hospital, Baqiyatallah University of Medical Sciences, Tehran, Iran; ^2^ Obstetrics and Gynecology Department, Baharloo Hospital, Tehran University of Medical sciences, Tehran, Iran; ^3^ School of Medicine, Tehran University of Medical Sciences, Tehran, Iran; ^4^ Baharloo Hospital, Department of Pediatrics, Tehran University of Medical Sciences, Tehran, Iran

**Keywords:** Iron Deficiency, Anemia, Pregnancy, Serum Ferritin, Serum Iron, TIBC

## Abstract

**Background:**

Iron deficiency during pregnancy poses a significant risk to maternal and fetal health, especially among anemic women whose physiological demand for iron increases drastically. This study aimed to evaluate the serum iron profile of non-anemic pregnant women at the time of delivery and to identify associated demographic and clinical factors.

**Materials and Methods:**

Conducted at Baharloo Hospital between 2022 and 2023, this cross-sectional study involved pregnant women who met the primary inclusion criteria of being non-anemic in the first and second trimesters and were admitted for scheduled labor. Serum ferritin, serum iron, and total iron-binding capacity (TIBC) were quantified, alongside comprehensive analysis of hemoglobin (HB) levels and red blood cell (RBC) indices. Data on demographic characteristics, and prenatal supplement usage were collected through medical records and analyzed using SPSS software.

**Results:**

This study of 120 pregnant women (mean age 28.85 ± 6.34 years, gestational age 39.21 ± 0.8 weeks) found that most participants regularly consumed iron (93.33%) and multivitamin supplements (81.67%), with average serum iron levels of 98.73 ± 20.7 μg/dL at 3rd trimester. Key correlations included negative associations between gestational age and maternal age (r=-0.26) and between ferritin and gestational age (r=-0.18), while hemoglobin and hematocrit levels were strongly positively correlated. Logistic regression identified lower second-trimester hemoglobin as protective against delivery-time anemia (OR=0.29), and lower second-trimester MCV significantly predicted iron-deficiency anemia (IDA) (OR=0.70), when adjusting for age, gestational age, BMI, supplements or iron usage, multiparity, and educational level.

**Conclusion:**

As iron and multivitamin supplementation did not significantly reduce anemia risk and it was basically related to second-trimester MCV and hemoglobin, higher thresholds of these markers should be assigned as the goal of anemia prevention programs for Iranian women.

## Introduction

Anemia is a major public health concern, predominantly more prevalent in low- and middle-income countries. Southeast Asia (48.15%), Africa (46.16%), and the Eastern Mediterranean (40.91%) are the regions with the highest prevalence of anemia globally, while the Americas (25.48%) observe the lowest [1]. This issue is so critical that reducing anemia rates is one of the six global nutrition targets set by the World Health Assembly, aiming for a 50% reduction in anemia among at-risk groups, such as women of reproductive age, by 2025 [2].

For women, the risk of iron deficiency and iron deficiency anemia increases during their reproductive years due to the increased iron requirements during pregnancy and the regular iron loss associated with menstruation [3]. In 2024, it is estimated that over 40% of pregnant women worldwide are anemic, with at least half of this anemia being due to iron deficiency [4]. The body’s need for iron increases dramatically during pregnancy as the mother’s blood volume increases and the fetus grows and develops [5]. During a full-term pregnancy, a pregnant mother needs approximately 1000 mg of iron, which is distributed as 300 mg for the fetus and placenta, and 500 mg for the growth of her red blood cells. The remaining iron is excreted from the body through the digestive system, skin, and urine [6]. One way to diagnose iron deficiency in pregnant women is to assess ferritin concentrations. A ferritin concentration of less than 30 μg/L during pregnancy indicates iron deficiency [7]. Hb and hematocrit (Hct) tests are also the most common measures used for screening patients for iron deficiency. Hb concentrations of less than 11 g/dL in the first and third trimesters, and 10.5 g/dL in the second trimester, indicate anemia [8].

Given iron’s role in numerous physiological processes during pregnancy, maintaining adequate iron balance among pregnant women is crucial. Studies show that iron imbalance during pregnancy, even in the absence of anemia, can be detrimental to both mother and child. Not only anemia but also iron deficiency during pregnancy is associated with preeclampsia, preterm birth, and even miscarriage [9][10]. Despite this, the primary focus of many studies has been on pregnant women who are anemic, whereas iron deficiency without anemia (IDWA) is at least twice as prevalent as iron deficiency anemia [11]. Even in high-income countries, up to 50% of non-anemic pregnant women have iron deficiency in the first trimester [12]. This means that many pregnant women may suffer from iron deficiency despite normal Hb levels. This can lead to more serious problems in the future, and the risk of missing a timely diagnosis of iron deficiency in these women increases the rate of complications during pregnancy and childbirth. On the other hand, iron deficiency without anemia is associated with symptoms such as fatigue, weakness, impaired function, restless legs syndrome, and irritability. These symptoms are all very common in pregnancy, which can lead to underestimating the diagnosis of iron deficiency in the pregnant mother and its consequences [13][14][15]. However, treating iron deficiency in these cases can lead to an improvement in patients’ symptoms. Iron deficiency without anemia should be corrected before childbirth to reduce the risk of postpartum anemia.

Considering the high prevalence of iron deficiency and the lack of recommendation for universal screening of ferritin levels to diagnose iron deficiency in pregnancy in many countries, including Iran, the present study investigated the serum iron profile in pregnant women who were non-anemic not only on admission but also during whole gestation and got admitted for normal vaginal delivery at Baharloo Hospital in 2022 and 2023. In this study, in addition to examining the iron profile, multiple factors such as maternal demographic status, ferritin levels, TIBC, and the use of iron and multivitamin supplements were also considered. This approach may provide a better understanding of the causes of hidden iron deficiency during pregnancy and its potential consequences on the health of mothers and infants.

## Materials and Methods

This cross-sectional study was conducted at Baharloo Hospital, focusing on non-anemic pregnant women during whole gestation admitted for delivery between 2021 and 2022. A convenience sampling method was employed to recruit a total of 120 participants who met the defined inclusion criteria: Pregnant women admitted for planned vaginal delivery (vaginal or cesarean) at term gestation (≥37 weeks), who were Non-anemic during first (Hb>11 g/dL, per WHO criteria) and second trimester (Hb>10.5 g/dL, per WHO criteria). Other inclusion criteria were: age> 18 years old, no evidence of clinical chorioamnionitis, absence of any signs or symptoms of clinical infection in other organs, no history of persistent vaginal bleeding or an episode of acute significant vaginal bleeding during pregnancy, no history of persistent bleeding or acute episode of significant bleeding from other body sites.

Women were excluded if they had pre-existing medical conditions affecting iron metabolism (e.g., chronic kidney disease, hemochromatosis, or inflammatory disorders), took more than one tablet of ferrous sulfate daily, or received intravenous iron during pregnancy, were in active labor (>4 cm cervical dilation) or had ruptured membranes at admission, manifested hypertensive disorders of pregnancy (e.g., preeclampsia or gestational hypertension), or presented with significant vaginal bleeding at delivery.

The sample size for this study was estimated at 96 participants based on a similar study [16], considering a confidence level of 95% and a margin of error of 10%. Ultimately, 120 eligible individuals were included in the study.

Data collection was performed using a structured questionnaire administered through direct interviews, alongside a comprehensive review of medical records. Demographic information recorded encompassed maternal age, gestational age, BMI, and the use of iron supplementation. Participants were included only if they had undergone Complete Blood Count (CBC) testing during both the first trimester (≤14 weeks) and second trimester (28-32 weeks) at the same study center, using standardized laboratory equipment and protocols. Serum iron profiles (ferritin, serum iron, and TIBC) were assessed from samples collected at delivery. Serum iron levels were quantified via colorimetric methods (mcg/dL), ferritin via ELISA, and TIBC was derived from serum iron and total iron capacity. All data were recorded on standardized forms to ensure consistency.

Iron-deficiency anemia (IDA) was defined as hemoglobin <12 g/dL with ferritin <30 ng/mL and TIBC >400 μg/dL, with higher thresholds of TIBC (standard cut-off for IDA is 450 μg/dL) to maintain the prevalence of IDA for statistical purpose.

Ethical approval for this study was granted by the Ethical Committee of Tehran University of Medical Sciences . Informed consent was also obtained from all participants.

### Data Analysis

The analyzed data utilized IBM SPSS (Version23, IBM, U.S.A) Statistics version 27. Descriptive statistics, including means and standard deviations (SD) for continuous variables, as well as frequencies and percentages for categorical variables, were employed to summarize participant demographics along with iron profile parameters. Correlation analyses were conducted to evaluate the relationships between various variables. Specifically, Pearson or Spearman correlation tests were utilized depending on the distribution of the data, focusing on the associations between serum ferritin levels and red blood cell indices across different trimesters of pregnancy. Additionally, logistic regression analyses were performed to determine predictors of serum iron levels and TIBC, incorporating covariates such as age, BMI, gestational age, and iron supplementation practices. Statistical significance was established at a level of P<0.05.

## Results

**Table:** Table1. Distribution of Demographic Characteristics and Laboratory Findings of Study Participants

Variable	Mean	Standard	Variable	Mean	Standard
Age (Years)		28.85	6.34	RBC (million/mm³)	First Trimester	4.1	0.37
Gestational Age (Weeks)		39.21	0.8		Second Trimester	4.3	0.33
Number of Pregnancies (Gravid)		2.11	1.19		At the Time of Delivery	4.44	0.37
Number of Miscarriages		0.325	0.663		First Trimester	38.00	2.47
Number of Live Children		0.775	0.902				
BMI (kg/m²)		30.45	4.84	HCT (%)	Second Trimester	35.84	2.46
	First Trimester	87.7	5.25				
MCV (µm³)	Second Trimester	87.37	5.68		At the Time of Delivery	37.51	2.35
	At the Time of Delivery	85.5	4.83				
	First Trimester	12.92	0.94	Ferritin Levels (µg/L)		30.2	23.7
HsB Levels (g/dL)	Second Trimester	11.92	0.80	Serum Iron Levels (µg/dL)		98.73	20.7
	At the Time of Delivery	12.39	0.88	TIBC (µg/dL)		410.56	54.04
	Illiterate	1	0.83	Iron Supplementation	Regular	112	93.33
	Diploma	52	43.33		Irregular	7	5.83
Education Level	Associate	5	4.17		None	1	0.83
	Bachelor's	17	14.17	Multivitamin Intake	Regular	98	81.67
	Post-Diploma	45	37.5		Irregular	11	9.17
					None	11	9.17

**Table:** Table2. Logistic Regression of Risk Factors for Delivery-Time Anemia (Hb<11 g/dL) *

**Predictor**	**Odds Ratio**	**95% CI**	**P-value**
**Iron Supplement (ref=No)**			
Regular	0.51	[0.05, 4.86]	.560
**Multivitamin (ref=None)**			
Irregular	2.37	[0.14, 40.93]	.554
Regular	3.85	[0.56, 26.62]	.172
BMI	0.91	[0.81, 1.03]	.130
Gestational age	1.18	[0.61, 2.26]	.624
Maternal age	0.91	[0.82, 1.01]	.070
Gravidity	1.14	[0.59, 2.23]	.693
Abortion history	1.17	[0.42, 3.22]	.767
**2nd Trimester Markers**			
RBC	1.04	[0.09, 12.79]	.973
**Hemoglobin**	**0.29**	**[0.09, 0.98]**	**.046**
Hematocrit	0.94	[0.61, 1.45]	.788
MCV	0.92	[0.78, 1.09]	.337
**1st Trimester Markers**			
RBC	0.16	[0.00, 7.86]	.361
Hemoglobin	0.47	[0.18, 1.23]	.123
Hematocrit	1.35	[0.74, 2.45]	.328
MCV	0.94	[0.73, 1.19]	.590

**Table:** Table3. Logistic Regression of Risk Factors for Iron-deficiency Anemia (IDA)

**Predictor**	**Odds Ratio**	**95% CI**	**P-value**
**Iron Supplement (ref=No)**			
Regular	0.91	[0.07, 12.69]	.945
**Multivitamin (ref=Irregular)**			
No	1.81	[0.22, 15.00]	.582
**BMI**	1.02	[0.86, 1.20]	.838
**Gestational age**	1.31	[0.54, 3.21]	.551
**Maternal age**	0.86	[0.73, 1.00]	.055
**Gravidity**	1.76	[0.66, 4.75]	.261
**Abortion history**	0.95	[0.21, 4.33]	.947
**2nd Trimester Markers**			
**RBC**	0.01	[0.00, 4.85]	.154
**Hemoglobin**	0.54	[0.08, 3.58]	.526
**Hematocrit**	1.52	[0.64, 3.58]	.340
**MCV**	**0.70**	**[0.53, 0.92]**	**.011**
**1st Trimester Markers**			
**RBC**	11.10	[0.03, 3654.98]	.416
**Hemoglobin**	0.41	[0.12, 1.39]	.150
**Hematocrit**	0.74	[0.35, 1.57]	.431
**MCV**	1.22	[0.89, 1.67]	.213

**Figure-1:**
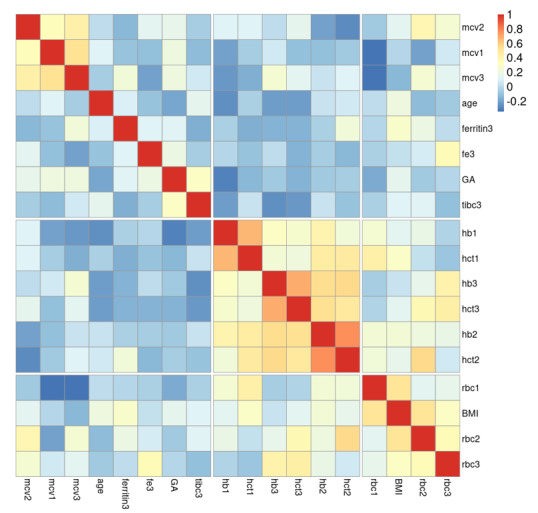
Figure 1. Correlogram of association of metric variables with each other, based on the Spearman correlation confidence, generated by the online app heatmapply.com

The study results indicated that among the 120 participants, the average maternal age and gestational age were 28.85 ± 6.34 years and 39.21 ± 0.8 weeks, respectively. The majority of pregnant women reported regular consumption of iron and multivitamin supplements. The study revealed an average serum iron level of 98.73 ± 20.7 µg/dL among the pregnant women. Further demographic characteristics and laboratory findings of the pregnant women are presented in Table-1. Further details comparing TIBC with body mass index, gestational age, maternal age, and iron supplement/tablet use are presented in Table-1. The participants' education levels were distributed as follows: 0.83% were illiterate, 43.33% held a diploma, 4.17% had an associate degree, 14.17% had a bachelor's degree, and 37.50% had post-diploma education. Regarding iron supplementation during pregnancy, 93.33% reported regular intake, 5.83% took it irregularly, and 0.83% did not take any. Similarly, multivitamin intake patterns showed that 81.67% took them regularly, 9.17% irregularly, and another 9.17% did not take any. The analysis revealed several statistically significant correlations among the variables. Gestational age (ga) showed a negative correlation with age (r=-0.26, P=0.004). Hemoglobin (hb1, hb2, hb3) and hematocrit (hct1, hct2, hct3) levels were strongly positively correlated with each other (r=0.41-0.85, P<0.001) and with red blood cell counts (rbc1, rbc2, rbc3) (r=0.24-0.58, P<0.01). Mean corpuscular volume (mcv1, mcv2, mcv3) exhibited negative correlations with rbc1/rbc2/rbc3 (r=-0.42 to -0.57, P<0.001) but positive correlations with each other (r=0.59-0.74, P<0.001). Ferritin3 was negatively associated with ga (r=-0.18, P=0.046), while fe3 (iron) correlated positively with age (r=0.22, P=0.016) and hb3 (r=0.20, P=0.029). BMI showed positive associations with rbc1 (r=0.19, P=0.042), hct1 (r=0.21, P=0.019), rbc3 (r=0.28, P=0.002), and hct3 (r=0.24, P=0.008). Total iron-binding capacity (tibc3) was inversely related to hb1 (r=-0.19, P=0.042) and hct1 (r=-0.22, P=0.016). No other significant correlations were observed as well as correlation of age or gestational age with CBC findings (Figure-1). A logistic regression examining risk factors for delivery-time anemia (Hb<11 g/dL) revealed that lower second-trimester hemoglobin was significantly protective (OR=0.29, 95% CI [0.09, 0.98], P=.046). Maternal age showed a marginally significant protective association (OR=0.91, P=.070). The model explained 27.1% of variance (pseudo R²=0.27) and was statistically significant (χ²(16) =38.36, P=.001). No significant associations were found for iron supplementation (OR=0.51, P=.560), multivitamin use (OR= 3.85, P=.172), or other hematologic parameters (Table-2). A logistic regression was performed to identify risk factors for iron-deficiency anemia (IDA), defined as hemoglobin <12 g/dL with ferritin <30 ng/mL and TIBC >400 μg/dL (13.33% prevalence, n=16/120). The model was statistically significant (χ²(15)=30.40, P=0.011, pseudo R²=0.34) and revealed that lower second-trimester MCV significantly predicted IDA (OR=0.70, 95% CI [0.53, 0.92], P=0.011). Maternal age showed a marginally protective trend (OR=0.86, P=0.055). No significant associations were found for iron supplementation (OR=0.91, P=0.945), multivitamins (OR=1.81, P=0.582), or other hematologic parameters (Table-3).

## Discussion

This cross-sectional study, conducted at Baharloo Hospital in Tehran, examined the serum iron profile in non-anemic pregnant women attending labor. The importance of iron during pregnancy cannot be denied, as it plays a crucial role in the physiological and metabolic processes essential for the health of both the mother and the developing fetus. Studies have shown that not only anemia but also iron deficiency during pregnancy is associated with preeclampsia, preterm birth, and even miscarriage [9][10]. The results of the present study indicated that 63.33% of the pregnant women who were non-anemic during their whole gestation had iron deficiency. Some studies also suggest that while a high percentage of pregnant women experience iron deficiency without anemia, it is often not accurately diagnosed because serum ferritin is not routinely measured [11][17]. This finding shows the complexity of iron metabolism during pregnancy, indicating that relying solely on hemoglobin and hematocrit levels as indicators of iron status may obscure the risks faced by women who are iron deficient but not yet anemic. A study conducted by Chibanda et al. in 2023 aimed to investigate the role of ferritin, Hepcidin, and cytokines in diagnosing iron deficiency anemia during pregnancy. The results of this research showed that the use of alternative and complementary tests for evaluating iron status could aid in a more accurate diagnosis. Moreover, this study showed the importance of hepcidin and inflammatory cytokines related to pregnancy in iron deficiency [18]. However, further research is needed in this area.

The results of the current study demonstrated no statistically significant correlation between serum ferritin and RBC-related indices in different trimesters of pregnancy, which aligns with the findings of Mirza Sultan et al. in 2020, indicating that serum ferritin had no correlation with any RBC-related indices [19].

This lack of correlation may be associated with the physiological and biochemical changes during pregnancy, which significantly affect ferritin levels and other blood indices. Given the considerable changes in blood parameters during this period, the importance of regular monitoring of iron profiles appears essential. This lack of correlation might suggest that relying solely on hemoglobin levels for diagnosing iron deficiency is insufficient, emphasizing the need for greater attention to ferritin as a primary marker of iron status. The results of this study indicated that the majority of participants reported regular consumption of iron and multivitamin supplements in both groups, those without deficiency and those with ferritin deficiency, with no statistically significant difference in the intake of these supplements between the two groups (P-value for iron supplement consumption = 0.478, P-value for multivitamin consumption = 0.312). Contrary to the findings of the present study, the study by Fite et al., which comprehensively examined iron status among pregnant women in eastern Ethiopia using serum ferritin concentration measurements, showed that more than half of these women suffered from iron deficiency, with significant factors such as low dietary diversity and insufficient meals associated with an increased risk of iron deficiency. In contrast, women receiving prenatal care had a lower risk of iron deficiency. These findings suggest that improving dietary diversity, along with iron supplements and enhanced nutritional counseling services, could positively impact reducing the burden of iron deficiency and improving pregnancy outcomes [20].

These findings may point to challenges in determining the appropriate dose and type of iron supplements, indicating a need for a more precise assessment of the type and dosage of supplements based on the individual needs of pregnant women.

The results of this study indicated a statistically significant correlation between serum iron and iron supplement consumption (P-value=0.013). This result shows the importance of appropriate iron absorption through specific nutritional interventions. Therefore, healthcare providers must consider individualized approaches tailored to each patient's needs when prescribing supplements. Indeed, there is a pressing need for a personalized approach to managing the iron status of pregnant women. In a study conducted by Burn et al. in 2023 at Yale University involving 5,054 pregnant women over eight years, the results indicated that the consumption of supplements or iron tablets by mothers did not have a significant impact on the occurrence of non-anemic iron deficiency. In other words, no significant difference was found between women who consumed these supplements and those who did not consume any supplements.

These findings somewhat align with the present study's results, particularly regarding the lack of observed differences between supplement intake and ferritin levels in pregnant women. Additionally, in Burn, et al.'s study, serum iron and TIBC showed no significant correlation with the intake of supplements or iron tablets. This scenario may indicate the depletion of iron reserves in the bodies of pregnant mothers and suggests that merely consuming supplements may not improve the iron status of mothers [21]. Conversely, the clinical trial by Karakoc, which focused on 264 anemic women in their second trimester of pregnancy, provided different results. This study, which aimed to compare the effects of daily iron tablets with ferrous fumarate tablets (containing 100 mg of elemental iron) taken for two months, showed that hemoglobin concentrations increased by 1.6 in the daily consumption group and by 1.4 in the other group (P-value:0.02). However, the prevalence of anemia after two months did not differ significantly between the two groups [22]. The reason for these differing results may relate to the heterogeneity of the study population and the varying types of supplements.

The results of this study indicated that no significant correlations were observed between serum iron and TIBC with maternal demographic features (BMI, gestational age, and maternal age).

The effects of these variables on iron status may be influenced by environmental and biochemical factors, necessitating further research in this domain. These findings indicate that solely relying on these demographic indices is insufficient for assessing iron status, showing the need to explore other influencing factors. In a study conducted by Aloy-Amadi et al. in 2020 aimed at investigating the levels of ferritin, serum iron, and TIBC in pregnant women, the results showed that ferritin and serum iron decreased with advancing gestational age, while TIBC increased with advancing pregnancy [23].

These results clearly show the challenges in diagnosing and managing iron deficiency in pregnant women. Despite iron supplement intake, many women may remain at risk of iron deficiency. Therefore, there is a need to examine and develop more precise and optimized treatment protocols to meet the iron needs of pregnant women.

This study shows the urgent need to enhance screening practices to identify and manage iron deficiency even in non-anemic pregnant women. Regular monitoring of iron status in prenatal care can serve as a vital tool for identifying women at risk of iron deficiency, allowing for timely interventions that may improve maternal and neonatal outcomes. Educating healthcare providers about the prevalence and potential risks associated with iron deficiency, especially in the absence of anemia, can enhance awareness and lead to improved screening practices. Future research should involve a broader population, taking into account various factors such as different socio-economic statuses, dietary practices, cultural contexts, and geographical backgrounds. Some maternal factors, like demographic characteristics, genetics, and lifestyle, influence the initial iron status of pregnant women [24].

The studies by Resseguier et al. (2022) [25], Zeng & He (2023) [26], Noshiro et al. (2022) [27], and El Ashiry et al. (2014) [28] collectively show the predictive role of first-trimester hemoglobin (Hb) and ferritin levels in identifying third-trimester anemia risk, though with varying optimal cutoff values. Resseguier et al. (2022) proposed a Hb cutoff of 120 g/L (specificity 87.5%) in a high-income setting, while Zeng & He (2023) reported a higher cutoff (128 g/L) in China, and Noshiro et al. (2022) identified 12.6 g/dL (126 g/L) as optimal in Japanese women. These discrepancies may reflect population-specific iron metabolism or dietary differences.

In contrast, El Ashiry et al. (2014) emphasized non-biochemical factors (e.g., multiparity, poor supplementation adherence) in an Egyptian cohort, where anemia prevalence was markedly higher (67%), suggesting socioeconomic and healthcare access disparities. Notably, while Resseguier et al. (2022) and Noshiro et al. (2022) found serum ferritin predictive, Zeng & He (2023) and our study showed Hb's superior predictive value, aligning with Noshiro et al.'s (2022) conclusion that Hb outperforms iron storage markers (ferritin, TIBC); while we did not have data of ferritin and TIBC at early pregnancy.

Our findings at Baharloo Hospital (2022-2023) diverged in key aspects: despite high iron/multivitamin supplementation rates (93.3%), second-trimester Hb and MCV were more critical predictors of delivery-time anemia than first-trimester values, contrasting with prior studies that focused on early-pregnancy markers. This suggests that in Iranian women, mid-pregnancy hematological indices may better reflect iron mobilization capacity than initial reserves. The negative correlation between gestational age and ferritin (r=-0.18) mirrors the physiological iron depletion observed in other studies, yet the lack of anemia reduction with supplementation challenges universal iron prophylaxis strategies. Unlike El Ashiry et al. (2014), we found no significant impact of parity or diet, possibly due to homogeneous supplementation practices. Our logistic regression identified lower second-trimester MCV as a specific predictor of iron-deficiency anemia (OR =0.70), showing erythrocyte indices’ role in risk stratification, a nuance absent in prior works. Collectively, these comparisons show that while Hb remains a robust predictor across populations, regional healthcare practices and genetic/nutritional factors necessitate tailored thresholds and timing for intervention.

## Conclusion

This study emphasizes the critical importance of assessing iron status, particularly serum ferritin levels, in non-anemic pregnant women. The findings indicate a significant prevalence of iron deficiency, even in the absence of anemia, showing its potential risks to both maternal and fetal health. Given that anemia risk was predominantly linked to second-trimester MCV and hemoglobin levels rather than iron/multivitamin supplementation, Iranian anemia prevention strategies should establish stricter cutoff targets for these hematological indices.

## Conflicts of Interest

The authors declare no conflicts of interest.
